# Targeting Neutrophil Extracellular Traps: A Novel Antitumor Strategy

**DOI:** 10.1155/2023/5599660

**Published:** 2023-11-09

**Authors:** Hao Zuo, Mengjie Yang, Qian Ji, Shengqiao Fu, Xi Pu, Xu Zhang, Xu Wang

**Affiliations:** ^1^Department of Radiation Oncology, Cancer Institute of Jiangsu University, Affiliated Hospital of Jiangsu University, Zhenjiang, Jiangsu, China; ^2^Jiangsu Key Laboratory of Medical Science and Laboratory Medicine, School of Medicine, Jiangsu University, Zhenjiang, Jiangsu, China; ^3^Department of Nursing, Nanjing University, Nanjing, Jiangsu, China; ^4^Department of Gastroenterology, The Affiliated Hospital of Jiangsu University, Zhenjiang, Jiangsu, China

## Abstract

The clinical efficacy of surgery, radiotherapy, and chemotherapy for cancer is usually limited by the deterioration of tumor microenvironment (TME). Neutrophil extracellular traps (NETs) are decondensed chromatin extruded by neutrophils and are widely distributed among various cancers, such as pancreatic cancer, breast cancer, and hepatocellular carcinoma. In the TME, NETs interact with stromal components, immune cells and cancer cells, which allows for the reshaping of the matrix and the extracellular environment that favors the initiation, progression, and metastasis of cancer. In addition, NETs impair the proliferation and activation of T cells and NK cells, thus producing a suppressive TME that restricts the effect of immunotherapy. A better understanding of the function of NETs in the TME will provide new opportunities for the prevention of cancer metastasis and the discovery of novel therapy strategies.

## 1. Introduction

Tumor microenvironment (TME) refers to the environment wherein tumor cells proliferate and undergo metastatic growth, including the extracellular matrix (ECM), microvasculature, inflammatory factors, and immune cells [1]. Due to the complexity of the components in the TME, the development and progression of tumor depends not only on tumor cells themselves but also on stromal cells and infiltrated immune cells, such as neutrophils. As the main innate immune cells for the defense against infection, neutrophils perform their functions by phagocytosis, the secretion of granules, the generation of reactive oxygen species (ROS), and NETosis (cell death-dependent release of neutrophil extracellular trap (NETs)) [2]. Neutrophils are involved in various stages of tumorigenesis and they can be divided into N1/N2 subtypes according to distinct functions and phenotypes [3]. Due to the rapid growth of tumors, local necrosis caused by insufficient blood supply or treatment leads to the release of large amounts of inflammatory factor and damage-associated molecular patterns (DAMPs) [4]. Neutrophils are recruited into the TME by inflammatory factors, including cytokines (CXCL1, IL-8/CXCL8, and CXCL12), complement (C3a, C5a), and lipid metabolites (LTB4) [5–9]. Then, N2 subtype tumor-associated neutrophils (TANs) are reeducated in the TME, inhibiting tumor-infiltrating CD8^+^ T cells by producing ROS, proteases, arginase, or expressing inhibitory immune checkpoint molecules, rather than killing tumor cells like N1 subtype neutrophils [10]. These neutrophil-chemotactic factors induce the formation of NETs to a certain extent. In addition, DAMPs produced by necrotic cells in the TME also induce toll-like receptor (TLR)-dependent NETosis [11]. The clinical treatments, such as radiotherapy and chemotherapy, may induce NETosis directly or indirectly through the abovementioned manners that promotes therapy resistance [12, 13].

As a complex composed of decondensed neutrophil chromatin and neutrophil-related proteases, NETs can promote tumor progression by inhibiting the proliferation, activation, and function of CD8^+^ T cells and NK cells [14]. NETs can carry neutrophil-derived programed cell death ligand-1 (PD-L1), which participate in immune regulation as an inhibitory component in the immune microenvironment [15]. Therefore, the inhibition of NETs can be a supplement to immunotherapy. In addition, NETs can also promote tumor growth by reshaping tumor cell metabolism and promote tumor metastasis by trapping cancer cells or directly binding to DNA receptors on tumor cells [16, 17]. Serum NET-specific DNA levels were closely related to the clinical stage of pancreatic cancer [18]. The existing findings on NETs and tumor have important implications for the clinic. The detection of NETs at the early stage of tumor development or premetastatic stage may help predict the severity of disease progression and the targeting of NETs with specific inhibitors may help better control the tumor and obtain a synergistic effect when combined with currently used treatments.

## 2. NETs in Tumor Development and Progression

### 2.1. NETs Facilitate Tumorigenesis

**Figure 1:**
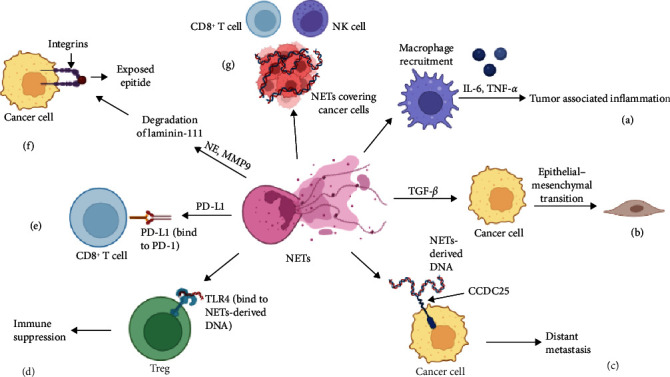
The protumoral effect of NETs. (a) NET-mediated macrophages provoked tumor-associated inflammation via IL-6 and TNF-*α*. (b) NET-derived TGF-*β* promotes the EMT of cancer cells. (c) NETs-derived DNA promotes cancer cell distant metastasis by binding DNA receptor CCDC25. (d) NETs induced naïve CD4^+^ T cells to differentiate into Tregs in a metabolism-dependent manner which suppressed Teffs. (e) The embedded PD-L1 in NETs inhibited the activation of T cells via PD-1. (f) NET-derived NE and MMP9 exposed a new epitope through the degradation of laminin-111, which awakened dormant cancer cells. (g) NET-insulated T cells and NK cells from cancer cells.

As the risk factor for hepatocellular carcinoma (HCC), nonalcoholic steatohepatitis (NASH) undergoes NET-mediated inflammation, which contributes to the development of HCC. Although the degradation of NETs via DNase cannot reverse steatosis of the liver, the researchers have observed that after stimulation with three free fatty acids that increase in NASH, neutrophils can generate an equivalent level of NETs. Subsequently, NETs promote the recruitment of macrophages (CD45^+^CD11b^+^F4/80^low^), which overwhelms Kupffer cells as the dominant inflammatory cells in the liver, thus upregulating interleukin-6 (IL-6) and tumor necrosis factor-*α* (TNF-*α*) and eliciting protumoral inflammation (Figure 1(a)). In addition, mice treated with DNase have shown an inhibited migration of neutrophils from the hepatic sinusoid to the liver lobule, thereby reducing the inflammation-induced initiation of HCC [19]. In brief, NET-induced recruitment of macrophages and production of inflammatory factors create a protumoral TME during the early stage of HCC.

In skin wounds, the keratinocyte-secreted high mobility group box-1 (HMGB1) has been shown to act as a DAMP by driving receptor interacting protein kinase-1 (RIPK1)-mediated apoptosis and necroptosis of neutrophils, leading to the enhanced release of TNF and the subsequent induction of NETs. The HMGB1-mediated NETs blocked wound healing which required a longer time to close cutaneous wounds. Moreover, the HMGB1-mediated formation of NETs facilitated wound-induced skin tumorigenesis, which could be suppressed via the injection of anti-TLR4, antireceptor for advanced glycation end products (RAGE), and Box A (which is HMGB1 antagonist) [20]. As a type of precancerous lesion, chronic skin inflammation induced by NETs can be a risk factor of skin tumorigenesis.

### 2.2. NETs Promote Tumor Progression

As a regulator of ECM homeostasis, tissue inhibitor of metalloproteinases-1 (TIMP-1) is involved in inflammation and tumor progression. The C-terminus of TIMP-1 binds to CD63 on neutrophils, which is upregulated by TNF-*α*; this mechanism activates the phosphorylation of extracellular signal regulated kinase (ERK), which phosphorylates the NADPH oxidase subunit p47. Then, the activated NADPH oxidase induces ROS-dependent NETosis [21]. The colocalization of TIMP-1 and NETs can be detected in the activating stromal areas, leading to a poor prognosis in pancreatic ductal adenocarcinoma (PDAC). Additionally, the combination of TIMP-1, NETs with CA19-9 show a more sensitive and specific prediction of survival when compared with a single marker [22].

ROS has been identified as a product of oxidative stress that is responsible for the formation of NETs by damaging the integrity of granules and nuclei [23]. In pancreatic cancer, cancer-associated fibroblasts (CAFs) secrete amyloid *β* in contact with neutrophils via CD11b, thus promoting ROS-mediated NETosis. CAFs support tumor growth via ROS-dependent and peptidylarginine deiminase 4 (PAD4)-dependent NETosis. As a result, NETs facilitate the proliferation of CAFs and endow CAFs with better expansion and contractility which contribute to matrix deposition [24].

In a spontaneous pancreatic cancer model, PAD4 expressed on neutrophils mediates the formation of NETs, thus upregulating the content of circulating neutrophil-derived DNA. Circulating DNA has been shown to elicit RAGE-dependent fibrosis of pancreatic stellate cells, which supported tumor growth by excluding T cells from the tumor cells [18].

In summary, NETs regulate the activation of signaling pathways in tumor cells or TME cells, which consequently promote tumor cell proliferation.

## 3. NETs in Tumor Metastasis

### 3.1. NETs Facilitate the Formation of the Premetastatic Niche

To reach the metastatic organ, tumor cells must overcome immune surveillance and adapt to the new environment. As “fertile soil” shaped by primary tumors through interactions with immune cells and stromal cells, the premetastatic niche supports tumor cell seeding, survival, and growth [25].

In breast cancer modeled by lung metastatic mouse mammary tumor virus (MMTV)-polyoma middle T (PyMT), breast cancer lung metastasis undergoes three stages, including adenoma (A), premetastasis (PM), and metastasis (M). The PM stage is characterized by inflammatory pathological changes, along with the infiltration of leukocytes. Compared with the A stage, there are more metastatic nodules in the lung at the PM stage, thus indicating a stronger proimplanting capacity, which is due to the NET-induced premetastatic niche. With the accumulation of Th2 cells in the PM niche, lung mesenchymal stromal cells (LMSCs) facilitate the formation of NETs via the C3-C3aR axis, which traps circulating tumor cells (CTCs) in the lung [26]. Likewise, in breast cancer, tumor-derived nicotinamide phosphoribosyltransferase (NAMPT) is involved in the generation of NAD^+^, thus supporting the NAD^+^-dependent deacetylation that regulates the transcription of lamin B receptor (LBR). LBR is regarded as an important component during oversegmentation of neutrophils. In conjunction with silent information regulator 1 (SIRT1)-mediated mitophagy, this effect can polarize neutrophils into aged neutrophils (Naged). In contrast to the classical citrullinated histone 3 (cit-H3) dependent NETosis, accumulated Naged in the lung constantly extrudes NETs via SIRT1-induced release of mitochondrial DNA (mtDNA). Afterward, NETs entrap the tumor cells, similar to the capture of microorganisms, thus promoting the formation of the premetastatic niche [16].

In the TME of ovarian cancer, IL-8, monocyte chemoattractant protein-1 (MCP-1) and growth-regulated oncogene *α* (GRO*α*) promote the recruitment of neutrophils from high endothelial venules (HEVs), according to the results of an array analysis. The H3-cit-positive Ly6G^+^ cells accumulate in the omentum which is a fatty tissue full of phagocytes, thus suggesting an infiltration of NETs. Moreover, there is a positive relationship between the formation of NETs and the degree of malignancy at stage Ⅰ/Ⅱ of cancer. As a type of negatively charged chromatin, the attachment of ovarian cells to NETs traps free cancer cells in the omentum, thus facilitating the formation of a premetastatic niche [27].

As the first site of metastasis, the immune microenvironment of lymph nodes can be changed by extracellular vehicles and be reshaped as a premetastatic niche [28]. Lymphatic endothelial cells expressed high level of CXCL8/2 via the uptake of tumor cell-derived extracellular vehicles, which induced neutrophil recruitment and NETosis, thereby promoting the formation of premetastatic niche [29].

Collectively, NETs adhere to and wrap up tumor cells due to their specific physicochemical properties, thus bringing CTCs to the metastatic organ.

### 3.2. NETs Induce Epithelial–Mesenchymal Transition (EMT)

The epithelial–mesenchymal transition (EMT) process contains a series of transitions in cell morphology and function that are influenced by endothelial cells, immune cells, and inflammatory cells via the snail family zinc finger 1 (Snail) and snail family zinc finger 2 (Slug) transcription factors. EMT contributes to the deficiency of cell polarity, the recombination of the cytoskeleton, and the loss of adhesion, which subsequently makes it easier for tumor cells to migrate and invade [30].

NETs induce EMT in breast cancer. NET-treated MCF7 cells exhibit higher zinc finger E-box binding homeobox 1 (ZEB1) and Snail mRNA levels, which regulate EMT. At the protein level, loss of epithelial marker E-cadherin (accompanied by the upregulation of mesenchymal markers such as N-cadherin, vimentin, and fibronectin) also demonstrates the presence of EMT. These NET-induced changes in gene and protein expression enable MCF7 cells to acquire fibroblast-like morphology and lose adhesion, thereby promoting metastasis. Additionally, NETs skew tumor cells to acquire stem–cell-like features by enhancing the expression of CD44 and reducing the expression of CD24 [31]. Moreover, NET-derived IL-1*β* mediates EGFR/ERK-dependent EMT in pancreatic cancer. The phosphorylation of EGFR significantly enhances the expression of the mesenchymal markers N-cadherin and vimentin, thus favoring EMT-induced metastasis [32].

Among the complications after surgery, postoperative abdominal infectious complications (AICs) are an obstacle to the survival of locally advanced gastric cancer (GC) [33]. AIC is known as a stimulant that enhances neutrophil motility and NETosis, which corresponds to the increasing IL-8 levels in the plasma. The infection-induced NETs rather than the neutrophil supernatant facilitate tumor cell proliferation and metastasis, which is dependent on the transforming growth factor-*β* (TGF-*β*)-mediated upregulation of N-cadherin and phosphorylated-smad family member 2/3 (*p*-Smad2/3), as well as the downregulation of *E*-cadherin (EMT marker) (Figure 1(b)). Moreover, the cluster formed by the attachment of GC cells to NETs supports CTC implantation [34]. Interestingly, EMT-induced metastasis and invasion in colon cancer cannot be diminished by heat, which further demonstrates that NETs-mediated EMT is based on the regulation of thermolabile proteins, such as vimentin, fibronectin, ZEB1, and Slug [35].

Mousset et al. [13] found that NETs mediated chemotherapy resistance via the induction of EMT in metastatic lungs in a breast cancer lung metastasis model. After the treatment of cisplatin or adriamycin/cyclophosphamide, increased neutrophils were detected in metastatic lungs instead of primary cancer due to the CXCL1/CXCL5 secretion. Dying cancer cells killed by chemotherapy released ATP, which led to the activation of NOD-like receptor family pyrin domain-containing 3 (NLRP3) and secretion of IL-1*β*. Then, IL-1*β*-induced-NETs acted as a scaffold to capture latent TGF-*β* via integrin-*α*v*β*1, cleaving latent TGF-*β* into an activated form to induce EMT.

NET-induced EMT also plays crucial role in pancreatic carcinogenesis by promoting the migration and invasion of tumor cells. In an obese model, Pdx1-Cre; LSL-KrasG12D^+/−^ (KC) mice accepted high-fat diet, which promoted the infiltration of neutrophils in pancreas, which can be induced to release NETs by visceral adipocytes. Then, NETs facilitated the EMT of pancreatic intraepithelial neoplasia cells via TLR4-dependent activation of IL-1*β* [36].

### 3.3. NET-Induced Distant Metastasis

NETs not only entrap CTCs as “nets” but also interact with tumor cells via receptors on the membrane. As a transmembrane protein with a N-terminus and C-terminus that are exposed to the outside surface of cancer cells, CCDC25 binds to NET-DNA (especially 8-OHdG-enriched DNA) via amino acids 21–25 (N-terminus). Furthermore, the C-terminus of CCDC25 interacts with integrin-linked kinase (ILK), which contributes to the recruitment of *β*-parvin, thus activating the *β*-parvin-RAC1-CDC42 cascade to facilitate the cytoskeleton rearrangement, and tumor cell proliferation and migration (Figure 1(c)) [17].

Although surgery is identified as an effective treatment for cancer, the inflammatory reaction elicited by surgery serves as a protumoral factor during distant metastasis [37]. Hepatic ischemia/reperfusion (I/R) injury increases the phosphorylation rate of ERK5 at Thr 218 and Thr 220 via TLR4 on platelets, thereby upregulating the expression of P70S6K and Rac1 which are necessary for integrin activation. Subsequently, platelets bind to the tumor cells via I/R-induced activation of integrin (GPIIb/IIIa) and P-selectin on platelets, thus leading to the formation of platelet–tumor cells aggregates. Additionally, the aggregates not only defend tumor cells from shear stress in the circulation but also attach I/R triggered NETs to CTCs which are deposited in the lung, thus facilitating distant metastasis [38].

As the main contributor of HCC-related mortality, lung metastasis is accompanied by the infiltration of neutrophils [39]. Yin et al. [40] found that metastatic HCC cell line SKHEP1 expressed lower histidine-rich glycoprotein (HRG), which was associated with lung metastasis. HRG interacted with the FC*γ*R1 of neutrophils, which inhibited the activation of PI3K and NF-*κ*B, in turn, suppressed IL-8-induced neutrophil recruitment and NETosis. Hence, HCC cells with lower expression of HRG underwent a NETs-mediated distant metastasis.

## 4. NETs and Immunosuppressive Tumor Microenvironment

### 4.1. NETs and Immunosuppressive Cells

The TME is characterized by the infiltration of immunosuppressive cells that are attracted by chemokines and cytokines and acquire immunosuppressive functions toward tumor cells. During this progression, neutrophils can be induced into a protumoral phenotype, thereby extruding NETs to interact with other immune cells and tumor cells.

Tregs serve as mediators to prevent the overactivation and self-injury of T cells, but they can encourage the immune escape of tumor cells under the influence of NETs. From a study of the microenvironment of NASH, Wang et al. [41] found that NETs interacted with naïve CD4^+^ T cells via TLR4, thus enhancing Treg function-associated genes, such as Tgfb1, Id3, Socs1, and Dusp4, and transforming naïve CD4 T cells into Tregs at the stage of differentiation. In contrast, effector T cell (Teff)-associated genes, such as Stat4, Il6st, Il1b, and Jak2, were found to be downregulated. As subsets with cytotoxicity, IFN-*γ*^+^CD4^+^ T cells and tumor-infiltrating CD8^+^ T cells were suppressed by Tregs in the hepatic environment (Figure 1(d)).

Th17 cells have been proved to facilitate tumor progression and therapy resistance via secretion of IL-17 and IL-22 [42, 43]. Similarly, NET-derived histone promoted naïve T cells differentiated into Th17 cells by TLR2/MyD88-dependent phosphorylation of STAT3, which activated transcription factor ROR*γ*t [44]. Another suppressive cell component in the TME is MDSCs. As previously reported, tumor-generated IL-8 can attract M-MDSCs and G-MDSC (granulocytic-MDSC) from peripheral blood via CXCR1 and CXCR2, thus facilitating the extrusion of NETs, which can be blocked by the specific CXCR1/2 inhibitor reparixin. Accordingly, the cells display different effects; specifically, when they are cocultured with T lymphocytes, M-MDSCs can inhibit the proliferation of both CD4^+^ and CD8^+^ T cells; and G-MDSCs can elicit NETosis in the presence of endotoxin-free IL-8. Ultimately, IL-8-induced NETs can entrap cancer cells and suppress T cells, thus shaping a protumoral environment [45].

In the case of APC mutation, defects in gut permeability provoke a bacterial and/or bacterial disorder in the intestinal system. Lipopolysaccharide (LPS) generated by bacteria acts as a stimulant to facilitate C3aR expression on neutrophils. As a crucial component during complement activation, C3a promotes the formation of NETs via the C3a-C3aR axis, which forms a hypercoagulation state, thus skewing neutrophils to a N2-like-low density neutrophil (LDN) phenotype. Clot-induced LDNs express high levels of arginase-1, which impairs the activation of T cells by exhausting L-arginine. Moreover, the expression of matrix metalloproteinase-9 (MMP-9) was also observed to be enhanced in LDNs. The abovementioned complement-mediated NETosis reshapes the immune phenotype of neutrophils, thus leading to a protumoral microenvironment [5]. Peng et al. [46] demonstrated that tumor cells disturbed the rigorous circadian fluctuation of neutrophils via the secretion of angiotensin Ⅱ, thus upregulating CXCR4 and downregulating CD62L on neutrophils. Due to the insufficient compensatory enhancement of macrophages, aged neutrophils cannot be cleared. Subsequently, the aged neutrophils showed increased ROS and released more MMP-9, inducing the activation of neutrophil elastase (NE) and most importantly promoting formation of NETs that mediate the tumor-associated inflammation and metastatic seeding in the microenvironment. The mutual interaction between immune cells and tumor reprograms the characteristics of neutrophils, and in turn, the tumor-educated neutrophils support the tumor to grow and metastasize via NETosis.

### 4.2. NETs and Tumor Metabolism

Tumor cells tend to utilize glycolysis rather than OXPHOS even under normal oxygen conditions, which are known as the Warburg effect [47]. The relationship between NETs and tumor metabolism has attracted much attention in recent years. Wang et al. [41] demonstrated that the depletion of regulatory T cells can effectively reduce the tumor burden and fibrosis in NASH. OXPHOS-associated genes are upregulated after interaction with NETs via TLR4 in naïve CD4^+^ T cells, which activates Tregs via the complex Ⅰ-dependent oxidation of NADH to NAD^+^. These changes in metabolism induce naïve T cells to differentiate into Tregs. Further studies found that NETs not only enhanced the oxygen consumption rate (OCR) but also reduced the extracellular acidification rate (ECAR) in CD4^+^ T cells after treatment. Metabolic remodeling impairs the cytotoxicity of Teffs, which deteriorates the microenvironment. The strong effect of NETs on metabolism may provide novel approaches to overcome immune suppression in the TME.

Another study demonstrated the profound mechanism of NETs in mitochondrial metabolism. Due to the hypoxic conditions in tumor, tumor cells express more neutrophil-associated chemokines (CXCL1, CXCL2, and CXCL5) and HMGB1 to rapidly recruit neutrophils. As the attractant of neutrophils, HMGB1 not only recruits neutrophils but also promotes the formation of NETs, which improve the expression of PGC1*α* via the TLR4-p38-PGC1*α* pathway. Ultimately, PGC1*α* acts as a mitochondrial biogenesis regulator to enhance the quality of mitochondria and the copy number of mtDNA, which facilitates OCR in tumor cells. Moreover, NETs maintain the mitochondrial homeostasis via dynamic fission, fusion, and mitophagy, which can be examined by the mitochondria-associated proteins DRP1 and MFN2. A set of NET-mediated mechanisms defend the mitochondria from detrimental impairment to provide energy for tumor growth [48]. Interestingly, tumor-secreted protein NAMPT acts as a rate-limiting enzyme in the upstream pathway of SIRT1, which restricts the OXPHOS of aged neutrophils (CD45^+^CD11b^+^Ly6G^+^CXCR4^+^CD62L^lo^) and prolongs the lifespan of neutrophils by mitophagy. Compared with nonaged neutrophils, Naged has a lower OCR, which contributes to the release of NETs via SIRT1-mediated permeability transition pore opening on the mitochondria. Subsequently, the NETs capture tumor cells, leading to the formation of a premetastatic niche in a metabolism-based manner [16].

In a metastasis model, NETs captured CTCs and upregulated hypoxia inducible factor-1*α* (HIF-1*α*) via ROS-dependent inhibition of proline hydroxylases. HIF-1*α* facilitated the stemness maintenance of CTCs and suppressed Teffs by increased expression of PD-L1 [49]. Although the metabolic changes in CTCs have not been explored, it is possible that NETs may promote glycolysis by HIF-1*α* in CTCs [50].

### 4.3. NETs and Immune Checkpoints

Immune checkpoints have been identified as negative costimulatory molecules in the immune cells [51]. In the TME of PDAC, IL-17 upregulates the expression of CXCL1, CXCL3, CXCL5, CSF3, and CCL20, which trigger the recruitment of neutrophils. Additionally, neutrophil-derived NETs impair the cytotoxicity of CD8^+^ T cells and reduce the number of CD8^+^ T cells via inhibition of CD8^+^ T-cell proliferation. Anti-IL-17 therapy was shown to effectively improve the spatial redistribution of the CD8^+^ T cells, thus making the cell cluster closer to tumor cells. Furthermore, the combination of anti-IL-17/IL-17R and anti-PD-1 decreased lactate levels of tumor, which reflected the growth inhibition of combined treatment at metabolic level. Interestingly, anti-IL-17/IL-17R, which reverses the NET-induced T-cell impairment, promotes the sensitivity of anti-CLTA4 in a CTL-dependent manner [52]. NETs isolated from patients with PDAC cleaved arginase 1 into different peptide fragments by cathepsin S, which made classical arginase 1 inhibitor lose efficacy. Canè et al. [53] designed neutralizing antibody of cleaved arginase 1, which rescued the inhibition of tumor-infiltrating lymphocytes when combined with Nivolumab and ipilimumab.

Kajioka et al. [54] utilized DNase Ⅰ (a NET inhibitor) to alleviate the NETs-mediated resistance to anti-PD-1. When combined with DNase Ⅰ, anti-PD-1 dramatically enhances the infiltration of CD8^+^ T cells into the TME and the expression of Prf1, granzyme B (GZMB), and IFN-*γ*, which indicates enhanced cytotoxicity. A similar effect can also be observed in PAD4-KO mice when treated with anti-PD-1.

Similarly, the NET-rich TME shows a suppressive function via the immune checkpoint during metastasis. NETs are the main source of PD-L1 (according to the staining of biomarkers). When cocultured with NETs, T cells express markers of exhaustion (including PD-1, Tim3, and Lag3) due to the embedded PD-L1 in the chromatin of NETs. Moreover, when cocultured with NETs, T cells exhibit reprogramed metabolic characteristics, including decreased mitochondrial function and decreased intake of glucose and fatty acids. Compared with the control, the group of PD-1-KO T cells or PD-L1-KO NETs downregulated TOX, which is a T-cell exhaustion-associated protein (Figure 1(e)). Treatment with DNase Ⅰ or PD-L1 blockade has been shown to abrogate the exhaustion of T cells in the TME [15].

Based on the current findings, NETs can induce T cells into an exhausted phenotype, which can be reversed by immune checkpoint inhibitors or combined with NETs blockade, representing a potential target for immunotherapy.

### 4.4. NET-Associated Inflammation

Chronic inflammation can promote enduring genetic and epigenetic changes, which protect tissue from inflammatory damage, favoring the malignant development of epithelial cells [55].

Stimulation of LPS or cigarette smoke extract simulates pulmonary inflammation, which triggers the formation of NETs. NET DNA acts as a bridge that tethers the neutrophil-associated proteases NE and MMP9 to laminin-111 in the ECM, thus leading to the cleavage of laminin-111. Additionally, thrombospondin-1 (TSP-1), which is a secreted protein that is involved in metastasis resistance, can also act as a substrate of NE and MMP9. As a result, the hydrolysis of laminin-111 exposes a new epitope, thus activating the signal pathway FAK/ERK/MLC2 via integrin *α*1*β*3 on cancer cells that awakens dormant cancer cells (Figure 1(f)) [56].

NET-induced inflammatory responses are involved in the progression of breast cancer. Due to the stimulation of NETs, inflammatory cytokines such as IL-1*β*, IL-6, and CXCL8 multiply in MCF7 cells and are accompanied by an increased expression of CXCR1, which mediates protumoral inflammation. Enzymes such as MMP2 and MMP9 dramatically increase in MCF7 cells when cocultured with NETs, which reshape the ECM, thus promoting metastasis [31].

In a study of HCC, Yang et al. [57] found that NET-mediated inflammation promotes tumor metastasis. Colocalized citrullinated histone 3 (H3cit) and myeloperoxidase-DNA (MPO-DNA) were detected in the serum of HCC patients which exhibited a high NETs level in plasma and a HCC-induced spontaneous NETosis. Subsequently, the HCC cells evoke sterile inflammation under the stimulation of NETs with the infiltration of the inflammatory mediators IL*α*/*β* and CSF-1, thus displaying the potential for angiogenesis. Cancer cells, entrapped by NETs, gain resistance to NET-induced cytotoxicity via the phosphorylation of NF-*κ*B and activation of the COX2. In particular, COX2 alleviates the cytotoxicity of NETs by TLR4/9, thus mediating aggressive HCC cell invasion based on the inflammatory response.

Previous studies have demonstrated that postoperative infection-induced sepsis can facilitate the formation of NETs which entrap circulating tumor cells, thus contributing to metastasis [58]. Similarly, operative stress, such as ischaemia‒reperfusion, can evoke inflammation-mediated NETosis in pancreatic ductal carcinoma. NETs entrap the tumor cells and can be observed under the electron microscope, thus leading to tumor cell extravasation into target organs. More significantly, neutrophils can secrete HMGB1 to combine with receptors on cancer cells (CD24 and TLR4), thus inducing EMT via the upregulation of vimentin (mesenchymal marker) and the downregulation of E-cadherin (epithelial maker). To block EMT-induced invasion, thrombomodulin (which inhibits DAMP-associated inflammation) is used to degrade HMGB1 in the presence of thrombin [59]. The use of thrombomodulin may be a feasible treatment to inhibit inflammation-induced NETosis and metastasis.

Metastatic colorectal cancer (CRC) cells expressed high fibroblast growth factor 19 (FGF), which induced the differentiation of hepatic stellate cells (HSCs) toward inflammatory cancer-associated fibroblasts (iCAFs) via the FGFR4-JAK2-STAT3 axis. Then, iCAFs promoted the infiltration of neutrophils by the secretion of inflammatory factors C5a and IL-1*β* [60]. C5a has been proved to facilitate the migration and invasion of neutrophils via the downregulation of *β*1 and *β*3 integrins in neutrophils [61]. Moreover, C5a induces NETosis via the activation of STAT3-ROS axis in the mitochondria of neutrophils [62]. The iCAFs-mediated NETosis made a metastasis-supported environment for CRC lung metastasis.

### 4.5. Immune Shield Function of NETs

Other than the common immune suppressive mechanisms mentioned above, NETs shield immune effector cells from target tumor cells in the TME, which is a similar function to “nets.” Cancer cells and endothelial cells are the primary cells that generate ELR^+^ CXCL1 and CXCL2 as agonists of CXCR1 and CXCR2, respectively, attracting neutrophils that can correspondingly extrude NETs. In vitro, when cocultured with NETs, the migration of T cells across the transwell to CCL5 is suppressed, which indicates poor T-cell motility. Under the intravital microscope, NETs cover and warp on the surface of cancer cells, through physical obstruction and negatively charged dsDNA-mediated electrostatic repulsion, thus decreasing the contact between NK cells, CD8^+^ T cells, and cancer cells (Figure 1(g)). Furthermore, it cannot be ignored that NETs contain the antibacteria components in neutrophils, such as NE, MPO, and other proteases, which impair the function of NK and T cells. Hence, the blockage of NETosis by reparixin (which is an antagonist of CXCR1 and CXCR2), DNase Ⅰ or PAD4 inhibitor exerts antitumor effects. Moreover, the blockage of NETs makes the tumor cells sensitive to the immune checkpoint inhibitors anti-PD-1 and anti-CLTA-4, thus suggesting a synergistic effect in immune therapy [14]. This study discovered a novel mechanism that differs from the classical interaction between NETs and immune cells. Based on this study, Cheng et al. [63] developed a hydrogel with tumor acidity neutralizer (mesoporous bioactive glass nanoparticles) and DNase to overcome NETs-induced inhibition of NK cells in HCC. The hydrogel effectively degraded NETs and neutralized acidic TME, which reduced the infiltration of immunosuppressive cells and enhanced the expression of NK-derived TNF-*α*, IFN-*γ*, and GZMB.

## 5. NETs as Novel Therapeutic Targets

Given the essential role of NETs in tumor progression, the potential of targeting NETs for therapeutic use with DNase or PAD4 inhibition has attracted more attention. Najmeh et al. [58] found that DNase can eliminate the surgical stress-elicited NETs adhesion to tumor cells, thus reducing NET-mediated metastasis. However, in another study, although DNase degraded NETs, the inhibition of NETs led to sepsis-induced death among nude mice that underwent caecal puncture. Given the antibacterial function of NETs, the author used a TGF-*β* inhibitor (LY 2157299) to block TGF-*β*-dependent NETs-induced EMT, thus effectively reducing metastasis without aggravating sepsis [34]. A previous study demonstrated the effect of PAD4; specifically, in the liver metastases of CRC, CRC cell-derived PAD4 induced the citrullination of collagen Ⅰ, thus promoting the metastasis of CRC [64]. Similarly, a PAD4 inhibitor (Cl-amidine/GSK484) was found to prevent NETosis by blocking the citrullination of histones, thus causing growth retardation in melanoma. Interestingly, PAD4 inhibitor treatment did not impact the recruitment of neutrophils but instead enhanced the expression of CD11b, CD18 (which are the active markers of neutrophils) and CD35, CD66b (which are the degranulation markers of neutrophils). Taken together, these results indicate that PAD4 inhibitors skew neutrophils toward a proinflammation and antitumoral phenotype [24]. A novel PAD4 inhibitor JBI-589 downregulated the expression of CXCR2 in neutrophils, which was accompanied by less TANs and more CD8^+^ T cells infiltration [65].

As phagocytes, neutrophils store proteases in the granules as critical components during tumor progression and invasion. Therefore, the targeting of the proteases also should be considered. The proteinase 3 inhibitor sivelestat diminishes the tumor-secreted protease cathepsin C-induced activation of PR3-IL-1*β*-NF-*κ*B-mediated recruitment of neutrophils and NETosis, thus further inhibiting NET-dependent colonization of tumor cells [66]. Treatment with MMP inhibitor (SB3-CT) and NE inhibitor (sivelestat) abrogates NET-mediated ECM remodeling, thus inhibiting the awakening of dormant cancer cells [56]. Moreover, the inhibition of NETs via NE inhibitor (GW311616A) or DNase alleviates HMGB1-TLR4-dependent NET-mediated resistance to radiation therapy [12]. The protease of NETs can also be specific detection indexes for NETs quantification. Cheng et al. [67] designed a tandem-locked NETosis reporter that activates fluorescence signals only in the presence of both NE and cathepsin G, which predicted the prognosis of immunotherapy.

Interestingly, targeting NETs displays synergistic effects when combined with immune checkpoint inhibitors (ICIs). As mentioned above, the blockade of IL-17, which recruits neutrophils, achieved better results when combined with anti-PD-1. The combination of ICIs (anti-PD-1 and anti-CTLA4) with a PAD4 inhibitor (GSK484) or DNase can reduce the lung metastasis of 4T1 cells [52]. To overcome the impairment of defense against infection caused by DNase, Chen et al. [68] designed a nanoplatform with broad-spectrum activity for the targeted delivery of DNase under laser irradiation. The irradiation-mediated release of DNase dramatically diminished NETs in the tumor, thus enhancing the sensitivity of anti-PD-1.

Finally, the routine treatments, including surgery, radiotherapy, and chemotherapy, tend to promote the recruitment of neutrophils and NETosis. Surgery-induced I/R promoted hypoxia-induced neutrophils accumulation and NETosis, which facilitated liver metastases via HMGB1-TLR9 pathway [69]. Chemotherapy-induced CXCL1/CXCL5 recruited neutrophils in the TME, which released NETs by NLRP3 inflammasome-derived IL-1*β* [13]. Nolan et al. [70] found that radiation promoted the infiltration of neutrophils in lung, which led to the metastasis of breast cancer. The recruitment of neutrophils could be induced by necrosis-mediated RIP1/RIP3/MLKL/JNK/IL8 signal pathway after radiation [71]. Neutrophils can mediate the resistance to radiotherapy via the activation of MAPK, which could be reversed by the depletion of neutrophils [72]. Radiotherapy-induced inflammation recruited PMN-MDSCs to the TME, which triggered NETosis via HMGB1-TLR4 pathway and endowed bladder cancer with resistance to radiotherapy [12]. Surgery and postsurgical radiotherapy frequently lead to locoregional failure since the tumor cells left outside the surgical tumor margins and overcome radiation [73]. Circulating breast cancer cells expressed high ectonucleotide pyrophosphatase/phosphodiesterase 1 (ENPP1) after radiation, which increased haptoglobin expression. The secreted haptoglobin facilitated neutrophil infiltration via the overexpression of CCR2 and promoted NETosis, inducing the local recurrence after surgery and radiotherapy [74]. Therefore, PAD4 blockade or DNase can reverse NETs-mediated surgery, radiotherapy or chemotherapy treatment resistance, or achieve a synergistic effect. In addition, inhibiting neutrophil-associated chemokines such as IL-1*β*, CXCL1/CXCL5 restrains NETosis. Blocking toll like receptors such as TLR4, TLR9 not only inhibits toll like receptors-dependent NETosis but also suppresses toll like receptors-dependent NETs-tumor cells crosstalk.  Table 1 summarizes the mechanisms and functions of NETs as potential therapeutic targets.

## 6. Conclusion

NETs are critically involved in tumor development, progression, metastasis, and therapy resistance, which are reflected by the wide crosstalk between NETs and TME cells. The tumor environment recruits neutrophils and induces the release of NETs by secreting certain proteins, inflammatory factors, DAMPs, exosomes, and among others. NETs promote tumor cell growth through metabolic remodeling of tumor cells and the support the suppressive microenvironment by directly damaging the function of CD8^+^ T cells or inducing the differentiation of immunosuppressive T cells, trapping CTCs, and promoting premetastatic niche formation. Moreover, clinical evidence suggests that NETs lead to resistance to radiotherapy, chemotherapy, and immunotherapy, and their levels are related to disease progression and therapeutic outcome.

Therefore, targeting NETs may have potential diagnostic and therapeutic significance. Conventional methods for targeting NETs by PAD4 inhibitors or DNase could clear NETs but may inhibit the antibacterial action of NETs and lead to spesis. Targeting the downstream pathway of NETs or using nanomaterials to deliver NET-targeting agents have been proved to be effective without affecting host response to infection. Antibody-drug conjugate may improve the specificity of targeting NETs and combined therapy with other drugs may have synergistic therapeutic effect. In addition, it is of great significance to analyze the levels of serum NETs, which may reflect the disease severity and predict prognosis. Furthermore, G-CSF is commonly used to treat radiotherapy and chemotherapy-induced neutropenia. Given that neutrophil recruitment and NETs may cause radiotherapy and chemotherapy resistance, it should be ascertained whether the clinical application of G-CSF would lead to NET-induced tumor recurrence or not.

Recently, a study found that melatonin promoted the infiltration of N1 subtype neutrophils by CXCL2, which killed pancreatic cancer cells by NET-derived ROS, indicating the antitumor effect of NETs [75]. It is unclear whether NETs from different subtypes of neutrophils have different effects on tumors, but it has been proved that N1 neutrophils exhibit higher levels of ROS and oxidative burst compared with N2 neutrophils [76]. If NETs derived from N1 subtype neutrophils kill tumor cells via higher level of ROS, repolarizing neutrophils toward N1 subtype and inducing NETs release may become an antitumor approach. Therefore, a further understanding of the biological roles and molecular mechanisms of NETs in tumor will help develop novel therapeutic approach design.

## Figures and Tables

**Table 1:** NETs as novel therapeutic targets.

Mechanisms	Targets	Drugs	Functions	References
Inhibits NETosis	PAD4	Cl-amidine/GSK484	Inhibits NET-induced tumor growth	[24]
Inhibits NET-induced EMT	TGF-*β*	LY 2157299	Reduces NET-induced metastasis	[34]
Inhibits NET-mediated ECM remodeling	MMP, NE	SB3-CT, sivelestat	Inhibits NET-induced awakening of dormant cancer cells	[56]
Inhibits NET-induced activation of TGF-*β*	MMP9	SB3-CT	Combined with cisplatin to overcome NET-induced chemotherapy resistance	[13]
Inhibits TLR4-dependent NETosis	HMGB1	Glycyrrhizin	Combined with radiotherapy to overcome NET-induced radiotherapy resistance	[12]
Inhibits ENPP1-induced NETosis	ENPP1	CM3163		[74]
Degrades NETs	NET-derived DNA	DNase	Combined with ICIs to overcome NET-induced T-cells inhibition	[52]
Inhibits NET-derived arginase 1	Arginase 1	Antiarginase 1		[53]

## Abbreviations

AICs: Abdominal infectious complications
*α*-SMA: *α*-smooth muscle actin
CAFs: Cancer-associated fibroblasts
Cit-H3: Citrullinated histone
3CRC: Colorectal cancer
CTCs: Circulating tumor cells
DAMPs: Damage-associated molecular patterns
ECAR: Extracellular acidification rate
ECM: Extracellular matrix
EMT: Epithelial–mesenchymal transition
ENPP1: Ectonucleotide pyrophosphatase/phosphodiesterase
1ERK: Extracellular signal regulated kinase
FGF: Fibroblast growth factor 19
GRO*α*: Growth-regulated oncogene
GZMB: Granzyme B
HCC: Hepatocellular carcinoma
HEVs: High endothelial venules
HIF-1*α*: Hypoxia inducible factor-1*α*
HMGB1: High mobility group box-1
HRG: Histidine-rich glycoproteini
CAFs: Inflammatory cancer-associated fibroblasts
ICIs: Immune checkpoint inhibitors
I/R: Ischemia/reperfusion
IL-6: Interleukin-6
LBR: Lamin B receptor
LDN: Low density neutrophil
LMSCs: Lung mesenchymal stromal cells
LPS: Lipopolysaccharide
MCP-1: Monocyte chemoattractant protein-1
MDSCs: Myeloid-derived suppressor cells
M-MDSCs: Monocytic myeloid-derived suppressor cells
MMP9: Matrix metalloproteinase-9
MMTV-PyMT: Mouse mammary tumor virus-polyoma middle T
MPO-DNA: Myeloperoxidase-DNA
MtDNA: Mitochondrial DNA
Naged: Aged neutrophils
NAMPT: Nicotinamide phosphoribosyltransferase
NASH: Nonalcoholic steatohepatitis
NE: Neutrophil elastase
NETs: Neutrophil extracellular traps
NETosis: Cell death-dependent release of NETs
NF-*κ*B: Nuclear factor kappa-B
NLRP3: NOD-like receptor family pyrin domain-containing 3
OCR: Oxygen consumption rate
OXPHOS: Oxidative phosphorylation
PAD4: Peptidylarginine deiminase 4
PDAC: Pancreatic ductal adenocarcinoma
PD-L1: Programmed cell death ligand-1
P-Smad2/3: Phosphorylated-smad family member 2/3
RAGE: Receptor for advanced glycation end products
RIPK1: Receptor interacting protein kinase-1
ROS: Reactive oxygen species
SIRT1: Silent information regulator 1
Slug: Snail family zinc finger 2
Snail: Snail family zinc finger 1
TANs: Tumor-associated neutrophils
Teff: Effector T cell
TGF-*β*: Transforming growth factor-*β*
TIMP-1: Tissue inhibitor of metalloproteinases-1
TLR: Toll-like receptor
TME: Tumor microenvironment
Tregs: Regulatory T cells
TSP-1: Thrombospondin-1
TNF-*α*: Tumour necrosis factor-*α*
ZEB1: Zinc finger E-box binding homeobox 1.

## References

[1] de Visser K. E., Joyce J. A. (2023). The evolving tumor microenvironment: from cancer initiation to metastatic outgrowth. *Cancer Cell*.

[2] Papayannopoulos V. (2018). Neutrophil extracellular traps in immunity and disease. *Nature Reviews Immunology*.

[3] Fridlender Z. G., Sun J., Kim S. (2009). Polarization of tumor-associated neutrophil phenotype by TGF-*β*: “N1” versus “N2” TAN. *Cancer Cell*.

[4] Karsch-Bluman A., Benny O. (2020). Necrosis in the tumor microenvironment and its role in cancer recurrence. *Advances in Experimental Medicine and Biology*.

[5] Guglietta S., Chiavelli A., Zagato E. (2016). Coagulation induced by C3aR-dependent NETosis drives protumorigenic neutrophils during small intestinal tumorigenesis. *Nature Communications*.

[6] Ding P., Li L., Li L. (2020). C5aR1 is a master regulator in colorectal tumorigenesis via immune modulation. *Theranostics*.

[7] Ogawa R., Yamamoto T., Hirai H. (2019). Loss of SMAD4 promotes colorectal cancer progression by recruiting tumor-associated neutrophils via the CXCL1/8-CXCR2 axis. *Clinical Cancer Research*.

[8] Cheng Y., Li H., Deng Y. (2018). Cancer-associated fibroblasts induce PDL1+ neutrophils through the IL6-STAT3 pathway that foster immune suppression in hepatocellular carcinoma. *Cell Death & Disease*.

[9] Satpathy S. R., Jala V. R., Bodduluri S. R. (2015). Crystalline silica-induced leukotriene B4-dependent inflammation promotes lung tumour growth. *Nature Communications*.

[10] Hedrick C. C., Malanchi I. (2022). Neutrophils in cancer: heterogeneous and multifaceted. *Nature Reviews Immunology*.

[11] Huang H., Tohme S., Al-Khafaji A. B. (2015). Damage-associated molecular pattern-activated neutrophil extracellular trap exacerbates sterile inflammatory liver injury. *Hepatology*.

[12] Shinde-Jadhav S., Mansure J. J., Rayes R. F. (2021). Role of neutrophil extracellular traps in radiation resistance of invasive bladder cancer. *Nature Communications*.

[13] Mousset A., Lecorgne E., Bourget I. (2023). Neutrophil extracellular traps formed during chemotherapy confer treatment resistance via TGF-*β* activation. *Cancer Cell*.

[14] Teijeira A., Garasa S., Gato M. (2020). CXCR1 and CXCR2 chemokine receptor agonists produced by tumors induce neutrophil extracellular traps that interfere with immune cytotoxicity. *Immunity*.

[15] Kaltenmeier C., Yazdani H. O., Morder K., Geller D. A., Simmons R. L., Tohme S. (2021). Neutrophil extracellular traps promote T cell exhaustion in the tumor microenvironment. *Frontiers in Immunology*.

[16] Yang C., Wang Z., Li L. (2021). Aged neutrophils form mitochondria-dependent vital NETs to promote breast cancer lung metastasis. *Journal for Immunotherapy of Cancer*.

[17] Yang L., Liu Q., Zhang X. (2020). DNA of neutrophil extracellular traps promotes cancer metastasis via CCDC25. *Nature*.

[18] Miller-Ocuin J. L., Liang X., Boone B. A. (2019). DNA released from neutrophil extracellular traps (NETs) activates pancreatic stellate cells and enhances pancreatic tumor growth. *OncoImmunology*.

[19] van der Windt D. J., Sud V., Zhang H. (2018). Neutrophil extracellular traps promote inflammation and development of hepatocellular carcinoma in nonalcoholic steatohepatitis. *Hepatology*.

[20] Hoste E., Maueröder C., van Hove L. (2019). Epithelial HMGB1 delays skin wound healing and drives tumor initiation by priming neutrophils for NET formation. *Cell Reports*.

[21] Hakkim A., Fuchs T. A., Martinez N. E. (2011). Activation of the Raf-MEK-ERK pathway is required for neutrophil extracellular trap formation. *Nature Chemical Biology*.

[22] Schoeps B., Eckfeld C., Prokopchuk O. (2021). TIMP1 triggers neutrophil extracellular trap formation in pancreatic cancer. *Cancer Research*.

[23] Brinkmann V., Zychlinsky A. (2007). Beneficial suicide: why neutrophils die to make NETs. *Nature Reviews Microbiology*.

[24] Munir H., Jones J. O., Janowitz T. (2021). Stromal-driven and amyloid *β*-dependent induction of neutrophil extracellular traps modulates tumor growth. *Nature Communications*.

[25] Houg D. S., Bijlsma M. F. (2018). The hepatic pre-metastatic niche in pancreatic ductal adenocarcinoma. *Molecular Cancer*.

[26] Zheng Z., Li Y.-N., Jia S. (2021). Lung mesenchymal stromal cells influenced by Th2 cytokines mobilize neutrophils and facilitate metastasis by producing complement C3. *Nature Communications*.

[27] Lee W., Ko S. Y., Mohamed M. S., Kenny H. A., Lengyel E., Naora H. (2019). Neutrophils facilitate ovarian cancer premetastatic niche formation in the omentum. *Journal of Experimental Medicine*.

[28] García-Silva S., Benito-Martín A., Nogués L. (2021). Melanoma-derived small extracellular vesicles induce lymphangiogenesis and metastasis through an NGFR-dependent mechanism. *Nature Cancer*.

[29] Su X., Brassard A., Bartolomucci A. (2023). Tumour extracellular vesicles induce neutrophil extracellular traps to promote lymph node metastasis. *Journal of Extracellular Vesicles*.

[30] Nieto M. A., Huang R. Y.-J., Jackson R. A., Thiery J. P. (2016). EMT: 2016. *Cell*.

[31] Martins-Cardoso K., Almeida V. H., Bagri K. M. (2020). Neutrophil extracellular traps (NETs) promote pro-metastatic phenotype in human breast cancer cells through epithelial-mesenchymal transition. *Cancers*.

[32] Jin W., Yin H., Li H., Yu X. J., Xu H. X., Liu L. (2021). Neutrophil extracellular DNA traps promote pancreatic cancer cells migration and invasion by activating EGFR/ERK pathway. *Journal of Cellular and Molecular Medicine*.

[33] Wang S., Xu L., Wang Q. (2019). Postoperative complications and prognosis after radical gastrectomy for gastric cancer: a systematic review and meta-analysis of observational studies. *World Journal of Surgical Oncology*.

[34] Xia X., Zhang Z., Zhu C. (2022). Neutrophil extracellular traps promote metastasis in gastric cancer patients with postoperative abdominal infectious complications. *Nature Communications*.

[35] Stehr A. M., Wang G., Demmler R. (2022). Neutrophil extracellular traps drive epithelial–mesenchymal transition of human colon cancer. *The Journal of Pathology*.

[36] Wang G., Gao H., Dai S. (2023). Metformin inhibits neutrophil extracellular traps-promoted pancreatic carcinogenesis in obese mice. *Cancer Letters*.

[37] Cools-Lartigue J., Spicer J., McDonald B. (2013). Neutrophil extracellular traps sequester circulating tumor cells and promote metastasis. *Journal of Clinical Investigation*.

[38] Ren J., He J., Zhang H. (2021). Platelet TLR4-ERK5 axis facilitates NET-mediated capturing of circulating tumor cells and distant metastasis after surgical stress. *Cancer Research*.

[39] Li P., Lu M., Shi J. (2020). Lung mesenchymal cells elicit lipid storage in neutrophils that fuel breast cancer lung metastasis. *Nature Immunology*.

[40] Yin Y., Dai H., Sun X. (2023). HRG inhibits liver cancer lung metastasis by suppressing neutrophil extracellular trap formation. *Clinical and Translational Medicine*.

[41] Wang H., Zhang H., Wang Y. (2021). Regulatory T-cell and neutrophil extracellular trap interaction contributes to carcinogenesis in non-alcoholic steatohepatitis. *Journal of Hepatology*.

[42] Perez L. G., Kempski J., McGee H. M. (2020). TGF-*β* signaling in Th17 cells promotes IL-22 production and colitis-associated colon cancer. *Nature Communications*.

[43] Peng D. H., Rodriguez B. L., Diao L. (2021). Th17 cells contribute to combination MEK inhibitor and anti-PD-L1 therapy resistance in KRAS/p53 mutant lung cancers. *Nature Communications*.

[44] Wilson A. S., Randall K. L., Pettitt J. A. (2022). Neutrophil extracellular traps and their histones promote Th17 cell differentiation directly via TLR2. *Nature Communications*.

[45] Alfaro C., Teijeira A., Oñate C. (2016). Tumor-produced interleukin-8 attracts human myeloid-derived suppressor cells and elicits extrusion of neutrophil extracellular traps (NETs). *Clinical Cancer Research*.

[46] Peng Z., Liu C., Victor A. R. (2021). Tumors exploit CXCR4^hi^ CD62L^lo^ aged neutrophils to facilitate metastatic spread. *OncoImmunology*.

[47] Koppenol W. H., Bounds P. L., Dang C. V. (2011). Otto warburg’s contributions to current concepts of cancer metabolism. *Nature Reviews Cancer*.

[48] Yazdani H. O., Roy E., Comerci A. J. (2019). Neutrophil extracellular traps drive mitochondrial homeostasis in tumors to augment growth. *Cancer Research*.

[49] Zhang Y., Wang C., Li W. (2022). Neutrophil cyto-pharmaceuticals suppressing tumor metastasis via inhibiting hypoxia-inducible factor-1*α* in circulating breast cancer cells. *Advanced Healthcare Materials*.

[50] Xu F., Huang M., Chen Q. (2021). LncRNA HIF1A-AS1 promotes gemcitabine resistance of pancreatic cancer by enhancing glycolysis through modulating the AKT/YB1/HIF1*α* pathway. *Cancer Research*.

[51] Sharma P., Siddiqui B. A., Anandhan S. (2021). The next decade of immune checkpoint therapy. *Cancer Discovery*.

[52] Zhang Y., Chandra V., Sanchez E. Riquelme (2020). Interleukin-17-induced neutrophil extracellular traps mediate resistance to checkpoint blockade in pancreatic cancer. *Journal of Experimental Medicine*.

[53] Canè S., Barouni R. M., Fabbi M. (2023). Neutralization of NET-associated human ARG1 enhances cancer immunotherapy. *Science Translational Medicine*.

[54] Zhang H., Wang Y., Onuma A. (2021). Neutrophils extracellular traps inhibition improves PD-1 blockade immunotherapy in colorectal cancer. *Cancers*.

[55] Navegantes K. C., de Souza Gomes R., Pereira P. A. Tártari, Czaikoski P. G., Azevedo C. H. M., Monteiro M. C. (2017). Immune modulation of some autoimmune diseases: the critical role of macrophages and neutrophils in the innate and adaptive immunity. *Journal of Translational Medicine*.

[56] Albrengues J., Shields M. A., Ng D. (2018). Neutrophil extracellular traps produced during inflammation awaken dormant cancer cells in mice. *Science*.

[57] Yang L.-Y., Luo Q., Lu L. (2020). Increased neutrophil extracellular traps promote metastasis potential of hepatocellular carcinoma via provoking tumorous inflammatory response. *Journal of Hematology & Oncology*.

[58] Najmeh S., Cools-Lartigue J., Rayes R. F. (2017). Neutrophil extracellular traps sequester circulating tumor cells via *β*1-integrin mediated interactions. *International Journal of Cancer*.

[59] Kajioka H., Kagawa S., Ito A. (2021). Targeting neutrophil extracellular traps with thrombomodulin prevents pancreatic cancer metastasis. *Cancer Letters*.

[60] Li C., Chen T., Liu J. (2023). FGF19‐induced inflammatory CAF promoted neutrophil extracellular trap formation in the liver metastasis of colorectal cancer. *Advanced Science*.

[61] Ortiz-Espinosa S., Morales X., Senent Y. (2022). Complement C5a induces the formation of neutrophil extracellular traps by myeloid-derived suppressor cells to promote metastasis. *Cancer Letters*.

[62] Chen Y., Li X., Lin X. (2022). Complement C5a induces the generation of neutrophil extracellular traps by inhibiting mitochondrial STAT3 to promote the development of arterial thrombosis. *Thrombosis Journal*.

[63] Cheng Y., Gong Y., Chen X. (2022). Injectable adhesive hemostatic gel with tumor acidity neutralizer and neutrophil extracellular traps lyase for enhancing adoptive NK cell therapy prevents post-resection recurrence of hepatocellular carcinoma. *Biomaterials*.

[64] Yuzhalin A. E., Gordon-Weeks A. N., Tognoli M. L. (2018). Colorectal cancer liver metastatic growth depends on PAD4-driven citrullination of the extracellular matrix. *Nature Communications*.

[65] Deng H., Lin C., Garcia-Gerique L. (2022). A novel selective inhibitor JBI-589 targets PAD4-mediated neutrophil migration to suppress tumor progression. *Cancer Research*.

[66] Xiao Y., Cong M., Li J. (2021). Cathepsin C promotes breast cancer lung metastasis by modulating neutrophil infiltration and neutrophil extracellular trap formation. *Cancer Cell*.

[67] Cheng P., He S., Zhang C., Liu J., Pu K. (2023). A tandem-locked fluorescent NETosis reporter for the prognosis assessment of cancer immunotherapy. *Angewandte Chemie International Edition*.

[68] Chen J., Hou S., Liang Q. (2022). Localized degradation of neutrophil extracellular traps by photoregulated enzyme delivery for cancer immunotherapy and metastasis suppression. *ACS Nano*.

[69] Tohme S., Yazdani H. O., Al-Khafaji A. B. (2016). Neutrophil extracellular traps promote the development and progression of liver metastases after surgical stress. *Cancer Research*.

[70] Nolan E., Bridgeman V. L., Ombrato L. (2022). Radiation exposure elicits a neutrophil-driven response in healthy lung tissue that enhances metastatic colonization. *Nature Cancer*.

[71] Wang Y., Zhao M., He S. (2019). Necroptosis regulates tumor repopulation after radiotherapy via RIP1/RIP3/MLKL/JNK/IL8 pathway. *Journal of Experimental & Clinical Cancer Research*.

[72] Wisdom A. J., Hong C. S., Lin A. J. (2019). Neutrophils promote tumor resistance to radiation therapy. *Proceedings of the National Academy of Sciences*.

[73] Krause M., Dubrovska A., Linge A., Baumann M. (2017). Cancer stem cells: radioresistance, prediction of radiotherapy outcome and specific targets for combined treatments. *Advanced Drug Delivery Reviews*.

[74] Ruiz-Fernández de Córdoba B., Moreno H., Valencia K. (2022). Tumor ENPP1 (CD203a)/haptoglobin axis exploits myeloid-derived suppressor cells to promote post-radiotherapy local recurrence in breast cancer. *Cancer Discovery*.

[75] Chan Y.-T., Tan H.-Y., Lu Y. (2023). Pancreatic melatonin enhances anti-tumor immunity in pancreatic adenocarcinoma through regulating tumor-associated neutrophils infiltration and NETosis. *Acta Pharmaceutica Sinica B*.

[76] Mihaila A. C., Ciortan L., Macarie R. D. (2021). Transcriptional profiling and functional analysis of N1/N2 neutrophils reveal an immunomodulatory effect of s100A9-blockade on the pro-inflammatory N1 subpopulation. *Frontiers in Immunology*.

